# Evaluating the treatment effectiveness of copper-based algaecides on toxic algae *Microcystis aeruginosa* using single cell-inductively coupled plasma-mass spectrometry

**DOI:** 10.1007/s00216-019-01933-9

**Published:** 2019-06-14

**Authors:** Xing Shen, Haiting Zhang, Xiaolong He, Honglan Shi, Chady Stephan, Hua Jiang, Cuihong Wan, Todd Eichholz

**Affiliations:** 1 Department of Chemistry, Missouri University of Science and Technology, 400 W 11th Street, Rolla, MO 65409 USA; 2 School of Life Sciences, Central China Normal University, Wuhan, 430079 Hubei China; 3 Center for Single Nanoparticle, Single Cell, and Single Molecule Monitoring (CS3M), Rolla, MO 65409 USA; 4 PerkinElmer Inc., Woodbridge, Ontario L4L 8H1 Canada; 5 Water and Sewer Department, City of Tulsa, Tulsa, OK 74103 USA; 6 Missouri Department of Natural Resources, Jefferson City, MO 65102 USA

**Keywords:** Single cell (SC)-ICP-MS, Harmful algal bloom, *Microcystis aeruginosa*, Copper-based algaecide, Microcystin-LR

## Abstract

Single cell-inductively coupled plasma-mass spectrometry (SC-ICP-MS) is an emerging technology. In this work, we have developed a novel SC-ICP-MS method to quantify metal ions in individual cells of a toxic cyanobacterial species, *Microcystis aeruginosa (M. aeruginosa)*, without complicated post-dosing sample preparation, and applied this method to study the treatment effectiveness of copper-based algaecides (cupric sulfate and EarthTec®) on the toxic algae *M. aeruginosa*. The developed SC-ICP-MS method uses new intrinsic metal element magnesium to determine real transport efficiency and cell concentration*.* The cell viability and microcystin-LR release by algaecide treatment were studied by flow cytometry and ultra-fast liquid chromatography-tandem mass spectrometry, respectively. The results showed that this novel method was very rapid, highly sensitive (detection limits of intracellular copper and magnesium were 65 ag/cell and 98 ag/cell, respectively), and reproducible (relative standard deviation within 12%). The algaecide effectiveness study further demonstrated that copper in the forms of cupric sulfate and copper-based algaecide EarthTec® successfully diminished *M. aeruginosa* populations. The higher the copper concentration used to treat the cells, the faster the speeds of copper uptake and cell lysis in the copper concentrations ranged from 0 to 200 μg/L of copper-based algaecide. The cells exhibit obvious heterogeneity in copper uptake. The result suggests that *M. aeruginosa* cells uptake and cumulate copper followed by cellular lysis and microcystin-LR release. These novel results indicated that though the copper-based algaecides could control this type of harmful algal bloom, further treatment to remove the released algal toxin from the treated water would be needed.

**Electronic supplementary material:**

The online version of this article (10.1007/s00216-019-01933-9) contains supplementary material, which is available to authorized users.

**Figure:**
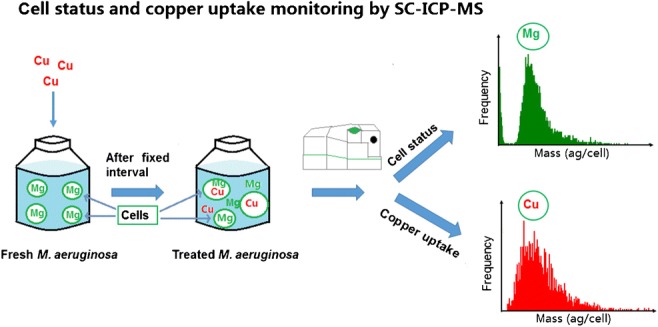
Graphical abstract

## Introduction

Harmful algal blooms (HABs) present a complex environmental challenge exacerbated by excessive nitrogen and phosphorus content in aquatic systems associated with agriculture practices [1] and climate change [2]. *Microcystis* blooms, in particular, have gained public attention owing to both the family of toxins, microcystins (MCs), and the global occurrence of such blooms. For example, Harke et al. [3] have reported *Microcystis* blooms in over 108 countries and the detection of MCs in 79 of those countries. Efforts to investigate interventions for *Microcystis* blooms have widely adopted *M. aeruginosa* as a model species owing to its significant toxicity compared with other *Microcystis* strains [4–8].

Interventions that have been suggested for *Microcystis* blooms have spanned mechanical, chemical, biological, genetic, and environmental approaches [9]. Mechanical solutions have involved clay flocculation [10], sonication and ultra-sound-enhanced coagulation [11], and artificial mixing [12], while biological approaches have proposed various organisms, particularly algaecidal microorganisms, as novel solutions to limit algae overgrowth [13–15]. Chemical methods have variously employed chemical reagents, such as copper-based algaecides [16–18], sodium percarbonate [19], sterol surfactants, sodium hypochlorite, and magnesium hydroxide, to control *Microcystis* blooms [20]. Among these interventional strategies, the use of cupric sulfate as an algaecide has advanced as an inexpensive and effective solution [18].

Although copper is an essential element for algae, elevated levels become cytotoxic by inhibiting photosystem II activity and electron transport [21], and can further damage cellular membranes [16]. As a result, *M. aeruginosa* has evolved at least four mechanisms to regulate intracellular copper, including (1) P-type ATPases that actively pump copper ions across the cell membrane; (2) copper chaperones that transport intracellular copper to copper-dependent proteins; (3) production of intracellular phytochelatin for copper detoxification; and (4) excretion of copper chelators such as phytochelatin [22]. For these reasons, there have been recent efforts to establish optimal copper concentrations for the effective treatment of *Microcystis* blooms and the control of its secondary pollution. Critical to these efforts has been the need to determine the cellular uptake of copper in its various proposed forms by *M. aeruginosa*.

Intracellular metal element quantification has conventionally involved sample digestion to determine total metal content from which an average cellular concentration may be derived. However, not only is this approach laborious and prone to sample contamination, it cannot provide intracellular concentrations for individual cells that are needed to construct mass distributions across an otherwise heterogeneous population. This limitation has resulted primarily from the inability of conventional analytical techniques to sample individual cells, such as total reflection X-ray fluorescence (TXRF), electrothermal atomic absorption spectrometry (ETAAS), inductively coupled plasma-mass spectrometry (ICP-MS), and inductively coupled plasma-atomic emission spectrometry (ICP-AES) [23]. However, this limitation has recently been overcome through the emergence of single cell-inductively coupled plasma-mass spectrometry (SC-ICP-MS) as a sensitive technique for intracellular metal quantification, down to the attogram (ag) per cell regime, to study gold nanoparticle biouptake by a freshwater nontoxic algae [24]. However, this emerging technology has not been used for toxic cyanobacteria analysis.

The principle of SC-ICP-MS technology has been introduced recently [25–27]. Briefly, single cell introduction into the ICP-MS system is achieved through the use of a modified nebulizer working in conjunction with a peristaltic pump that is used to deliver small volumes of cell suspension into the spray chamber. By optimizing cell concentration and flow rate, it allows individual cells to enter the plasma. Individual cells become ionized in the plasma as discrete plumes that are subsequently detected as pulsed signals by the mass spectrometer. The pulse signal intensity is proportional to the elemental mass in an individual cell whereas the pulse signal frequency relates to the cell concentration within the cell suspension. Moreover, the baseline signal in the absence of a pulse represents the extracellular concentration of the analyte within the cell suspension. In this way, the concentrations of cells containing detectable analyte, computed masses of the analytes in individual cells, mass distributions within the cell population, and extracellular analyte concentrations can all be quantified simultaneously. Li et al. [28] first introduced intact single cells into magnetic sector ICP-MS directly, and reported that the intact individual bacterium behaved like a large particle in the ICP-MS. In the following several years, SC-ICP-MS was proposed and applied for metal content analysis in multiple species, including bacteria [29, 30], algae [24, 26, 31], yeast [32], and human cancer cells [25, 33–39]. Due to the capability to sensitively and rapidly measure metal content within individual cells, SC-ICP-MS is expected to be a rapid developing powerful technology with an enormous potential for applications in drug development, heavy metal toxicity study, metallomics, and other life sciences and environmental researches.

Accordingly, the purpose of this study was to develop a new high-throughput SC-ICP-MS method to monitor cell status and quantify copper uptake and accumulation in a toxic alga *M. aeruginosa* following exposure to proposed copper-based algaecides, by using a commercially available instrument. This approach would enable improved characterization of copper-based algaecides and their underlying mechanisms at the single cell level in order to better control *Microcystis* blooms. The resulting method was then validated by assessing cell viability following exposure using flow cytometry and the release of microcystin-LR (MC-LR) using ultra-fast liquid chromatography-tandem mass spectrometry (UFLC-MS/MS).

## Materials and methods

### Reagents and chemicals

Elemental metal analytical standards were obtained from PerkinElmer Inc. (Shelton, CT, USA). Calibration standards were prepared from mixed standards of dissolved copper and magnesium, along with sterile modified BG-11 culture medium and 0.1 mM ethylenediaminetetraacetic acid (EDTA) to approximate sample matrices. The modified BG-11 medium was prepared by a fivefold dilution of the original BG-11 with ultrapure water in the absence of any manganese-, copper-, or magnesium-based compounds [40]. Ultrapure water (18.2 MΩ·cm) was produced by a Simplicity 185 water system from Millipore (Billerica, MA, USA). *M. aeruginosa* cells were diluted with sterile 0.1 mM EDTA (Sigma, St. Louis, MO, USA) in order to chelate copper present in solution and bound to cell surfaces [41]. This approach permitted direct analysis of the cells without a post-treatment washing process. A certified reference standard for drinking water (CRM-TMDW-A, from Charleston, SC, USA) was used to verify the accuracy of the calibration curve. Cupric sulfate stock solutions were prepared to a concentration of 100 μg copper per milliliter using sterile ultrapure water. EarthTec® is a widely used algaecide containing 19.8% cupric sulfate pentahydrate that was provided to the research team from a drinking water treatment plant in order to evaluate copper uptake in commercially used forms of copper-based algaecide. All bottles and tubes were soaked in 3% HNO_3_ overnight followed by three rinses with ultrapure water for cleaning purposes.

### Instruments

A PerkinElmer NexION 300D ICP-MS (Shelton, CT, USA) equipped with Syngistix Single Cell Application software was used for data collection and processing. The sample introduction system was equipped with a quartz single cell spray chamber, a Meinhard nebulizer, a platinum sampler, and skimmer cones. The spray chamber was regulated by a heater and thermometer at 29~32 °C in order to prevent condensation on the inner wall of the spray chamber. The sampling system was washed with 2% nitric acid followed by ultrapure water between samples. A BD AccuriTM C6 Cytometer (Ann Arbor, MI, USA) and SYTO® 9 green fluorescent nucleic acid stain and propidium iodide red fluorescent nucleic acid stain were used to assess cell viability by following the manufacture’s instruction. An UFLC-MS/MS device as Zhang et al. descripted [42] was used to analyze intra- and extracellular microcystin-LR content in the algal cell suspension after algaecide treatment.

### Cell culture

Unicellular cyanobacterium *M. aeruginosa* (UTEX LB 2388) was purchased from the Culture Collection of Algae at The University of Texas, Austin, USA, and cultured in BG-11 growth medium (Sigma-Aldrich, Saint Louis, MO, USA) as described by Ding et al. [43]. The late exponential phase was in the range of 14 to 22 days, which was determined by counting cells every 2 days after sub-culture.

Two batches of cells from different dates were used for cupric sulfate and EarthTec® algaecide treatment, respectively.

### SC-ICP-MS method development

The SC-ICP-MS method development was started by assessing cell integrity before and after sampling and nebulization at variable sampling flow rates (20 and 42 μL/min) and nebulization gas flow rates (0, 0.3, 0.5, and 0.7 L/min). All the samples were tested in triplicate. Visible intact cells were counted by a hemocytometer (Hausser Scientific) with an optical microscope (Olympus CH-2 CHT) before and after nebulization.

Next, the SC-ICP-MS methods to quantify metals in *M. aeruginosa* were developed. An intrinsic metal magnesium (the most abundant isotope ^24^Mg was monitored) in *M. aeruginosa* cells, which has a high-enough quantity in individual *M. aeruginosa* cells to be detected by SC-ICP-MS, was used to determine instrument transport efficiency (TE) and the cell status. The method was optimized with respect to dwell time, sampling flow rate, and cell concentration in order to maximize sensitivity. RF power and makeup gas flow rate were set at fixed values. The method quantification detection limits for extracellular copper (^65^Cu was selected as the quantitative isotope owing to interferences for ^63^Cu) and magnesium were determined from standard solutions using the method reported by Dan et al. [44]. The quantification detection limit for intact cell concentration was determined from the abundant intrinsic metal pulse signal frequencies using serial dilutions of fresh cell suspensions, a similar method with the nanoparticle concentration quantification detection limit determination [44], i.e., the detected cell concentration in good agreement with the prepared cell concentration. The detection limit for intracellular copper mass per cell was determined to be the lowest metal mass in the mass distribution histogram, equivalent to 3 times the standard deviation of background noise. All samples were measured in at least triplicate. A concentration of approximately 500,000 cells/mL of fresh *M. aeruginosa* cell suspension was used to determine the TE by monitoring the abundant intrinsic element ^24^Mg. This method should generate the most accurate TE for cells because the same cells were used under the same matrix.

### Algaecide treatment experiments

Cells were harvested in the late exponential phase, followed by centrifugation at 500*g* for 10 min. The supernatant was discarded, and the cell pellet was washed twice with sterile modified BG-11 medium by centrifugation at 500*g* for 5 min. The cell pellet was re-suspended in the modified BG-11 medium to constitute the cell stock standard suspension. Cell concentrations were determined with a hemocytometer. The cell stock suspension was diluted to 500,000 cells/mL with 0.1 mM EDTA, and the cell concentration in the diluted cell suspension was counted again to obtain the exact cell concentration for determination of TE for SC-ICP-MS analysis. TE was determined daily before each experiment. For algaecide treatment experiments, cell stock was diluted to 1,000,000 cells/mL with modified BG-11 medium, and cells were finally treated with cupric sulfate or EarthTec® at concentrations of 0, 30, 60, 100, and 200 μg/L copper, respectively.

At fixed time intervals, the concentrations of intact cells, extracellular copper and magnesium concentrations, and the concentrations of cells containing detectable levels of copper in control groups were quantified after a three-fold dilution of cell suspension with 0.1 mM EDTA. For the treatment groups, samples were diluted to reach a copper concentration of 10 μg/L based on the dosed copper concentration before SC-ICP-MS detection of the concentration of the cells containing detectable levels of copper and intracellular copper masses per cell, to minimize the impact of the extracellular copper signal on the pulsed cell signal. The matrix-matched dissolved copper and magnesium standard solutions were used to make calibration curves for intra- and extracellular copper and magnesium quantification. All the samples were analyzed immediately to avoid possible metal release or continuous uptake to ensure the accuracy of copper uptake quantification. At the same time, samples were also collected for cell viability detection by a flow cytometer and MC-LR detection by the UFLC-MS/MS analysis with the method described by Zhang et al. [42]. Because all samples need to be analyzed immediately by the SC-ICP-MS, flow cytometry, and UFLC-MS/MS methods, only selected treatments were duplicated (60 μg Cu/L of two copper-based algaecide treatments).

## Results and discussion

### SC-ICP-MS analysis method development

**Fig. 1:**
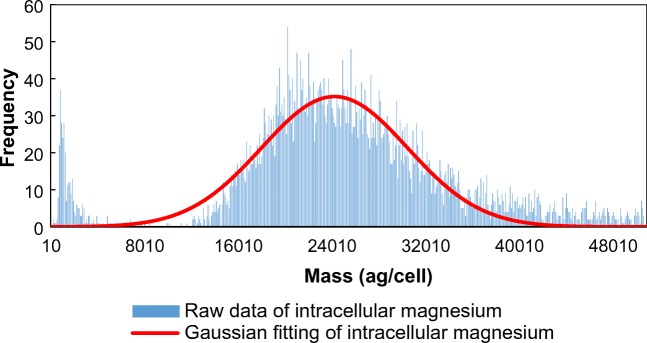
Intracellular magnesium mass distribution in fresh *M. aeruginosa* cells

TE refers to the ratio of analyte entering the plasma to the amount of analyte aspirated [45], and represents a key parameter for cell concentration quantification by SC-ICP-MS. Pace et al. [46] compared three methods of measuring TE of SP-ICP-MS technology for particle detection, the waste collection method, particle size method, and particle frequency method, finding that the waste collection method tended to overestimate TE, while the particle size and particle frequency methods tended to correctly estimate TEs. However, unlike SP-ICP-MS in which the particle integrity is maintained throughout the nebulization process, many cell types are prone to lysis during sample introduction. In this study, cells were collected after nebulization and the cell integrities were examined using a microscope to verify that the sample introduction system was operating as intended without damaging cells. These results, which have been shown in Supplementary Electronic Material (ESM) Fig. S1, indicated that *M. aeruginosa* cells were successfully entering the ICP-MS as intact cells following sampling and nebulization. Next, an intrinsic metal that physiologically accumulates in *M. aeruginosa* was selected to detect the intact individual cells into the ICP-MS system. Our initial assessment of common intrinsic metals found that intracellular magnesium was abundant in intact *M. aeruginosa* cells, as shown in Fig. 1, to be used for rapid cell concentration determination by SC-ICP-MS and TE detection. Therefore, intracellular magnesium in fresh cells could be monitored in order to determine real-time TE. This novel TE determination method using cells themselves by detecting the intrinsic metal in individual cells represents real cell status; thus, it should make more accurate quantification than the other methods. It should be noticed that the small peak at low masses might come from the old cell debrides containing a small amount of Mg. When we determined the intact cell concentrations, we excluded these small peaks.

TE was calculated automatically by Syngistix Single Cell Application software using the following equation (similar with the particle frequency method referenced above [46]):

$$ \eta =\frac{N}{C\times \left(Q\times T\right)}\times 100\%, $$

where *η* is TE, *N* is the number of peaks, *Q* is the sample flow rate, *T* is the scan time, and *C* is the cell concentration as determined by a hemocytometer. The mass spectrometer dwell time, sample flow rate, and cell concentration were all optimized to yield a high and stable TE. The optimization of these method parameters is shown in ESM Fig. S2 while the optimized parameters are summarized in Table 1. Fresh cell suspension at a cell concentration in the range of 5000 to 1,000,000 cells/mL was found optimum for TE detection; finally, 500,000 cells/mL of fresh cell suspension was used for TE determination. RF power and makeup gas flow rate were fixed, while nebulization gas flow rate and TE vary from day to day due to routine daily optimization and determination, respectively.

**Table 1:** Optimized SC-ICP-MS analysis method parameters

Parameter	Value
RF power (W)	1600
Nebulization gas flow rate (L/min)^a^	~ 0.5
Makeup gas flow rate (L/min)	0.7
Sample flow rate (μL/min)	21~22
Dwell time (μs)	100
Scan time (s)	100
Transport efficiency (%)^b^	45.56~63.65
Analyte	^65^Cu, ^24^Mg

^a^Parameter is re-optimized daily; ^b^Parameter is determined daily

The mixed standards of dissolved copper and magnesium were used to make all calibration curves. Quantification detection limits for extracellular copper, extracellular magnesium, and cell concentration were determined to be 1 μg/L, 0.2 μg/L, and 3000 cells/mL, respectively; quantification detection limits for intracellular copper and magnesium mass per cell were 65 ag/cell and 98 ag/cell, respectively (Table 2).

**Table 2:** Detection limits for selected extracellular metal concentrations, *M. aeruginosa* cell concentration, and selected intracellular metal masses per cell

Name	Detection limit	
	Concentration	Intracellular mass per cell
^65^Cu	1 μg/L	65 ± 7 ag
^24^Mg	0.2 μg/L	98 ± 12 ag
*M. aeruginosa*	3000 cells/mL	/

### Copper-based algaecide effectiveness assessment by SC-ICP-MS

**Fig. 2:**
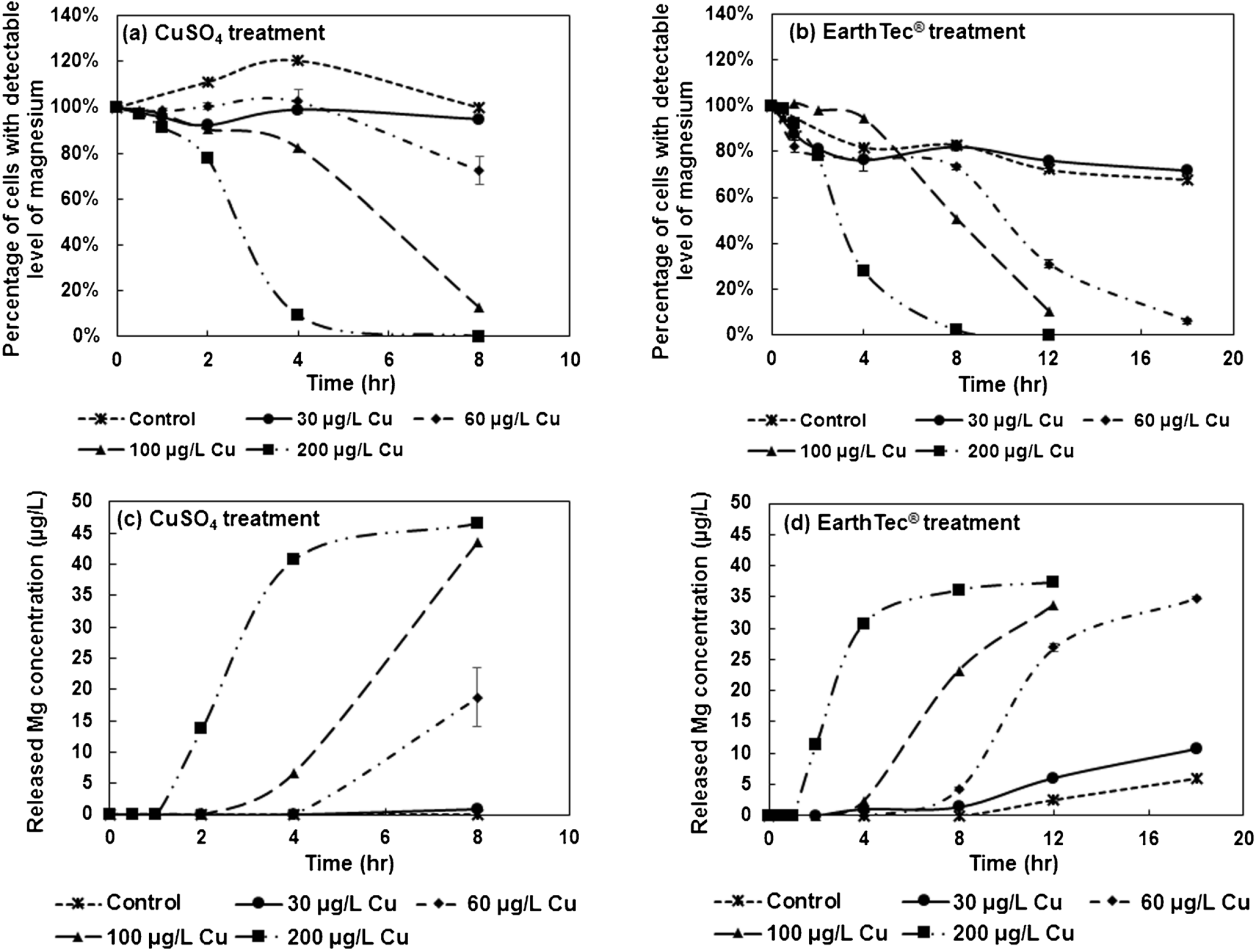
Percentage of cells with detectable levels of magnesium following exposure to (**a)** CuSO_4_ and (**b)** EarthTec® treatments over time, and extracellular magnesium concentrations following exposure to (**c)** CuSO_4_ and (**d)** EarthTec® over time using SC-ICP-MS

Given that an intrinsic magnesium pulse signal was detectable only in intact cells, evidenced by the agreement of SC-ICP-MS and flow cytometer detections, the loss of intracellular magnesium signals following exposure to the copper-based algaecides was attributed to cellular lysis causing magnesium to be released into the extracellular matrix. Significant levels of magnesium were not found in the algaecide reagents, modified BG-11 medium, and 0.1 mM EDTA solution. Hence, we monitored the variations of cell status in *M. aeruginosa* populations after algaecide treatment over time by quantifying intact cell concentrations and released magnesium ion concentrations. For each experiment, the calibration curve of dissolved magnesium generated high linearity (*R*^2^ > 0.98) and quality control water reference standard CRM-TMDW-A (High Purity, USA) was analyzed to make sure quality data was generated. Following exposure to cupric sulfate (0, 30, 60, 100, and 200 μg/L copper), the percentage of cells with detectable levels of magnesium did not change significantly in the 30 μg/L treatment group and decreased to 73%, 13%, and less than the detection limit in the 60 μg/L, 100 μg/L, and 200 μg/L treatment groups, respectively, after 8 h of treatment (Fig. 2a). Extracellular magnesium, indicative of lysis-induced metal release, increased following treatment for 4 h in the 60 μg/L treatment group, 2 h in the 100 μg/L treatment group, and 1 h in the 200 μg/L treatment group. Only slight increases in extracellular magnesium were observed in the 30 μg/L treatment group after treatment for 8 h (Fig. 2c). Similarly, the percentage of cells with detectable levels of magnesium decreased following treatment with EarthTec® for 8 h in the 60 μg/L treatment group, 4 h in the 100 μg/L treatment group, and 2 h in the 200 μg/L treatment group. Cells in the control group and the 30 μg/L treatment group started lysing following treatment with EarthTec® for 8 h, yet more than 70% of cells remained intact after treatment for 18 h (Fig. 2b). Extracellular magnesium was also found to increase after treatment with EarthTec® for 4 h in the 60 μg/L treatment group, 2 h in the 100 μg/L treatment group, and 1 h in the 200 μg/L treatment group. However, extracellular magnesium increased after treatment for 8 and 2 h in the control and the 30 μg/L treatment groups, respectively (Fig. 2d). The possible reason for the difference of magnesium release from the 30 μg/L treatment groups in cupric sulfate and EarthTec® treatments was that the cells were from different batches. These results suggested that cell lysis and magnesium release occurred in response to higher doses of copper in the forms of cupric sulfate and EarthTec® while minimal effectiveness was observed in the 30 μg/L treatment groups. In addition, extracellular magnesium increased faster in *M. aeruginosa* cells that were treated with cupric sulfate compared with EarthTec®, indicating cupric sulfate functions faster than EarthTec®.

### Copper-based algaecide effectiveness validated by flow cytometer

**Fig. 3:**
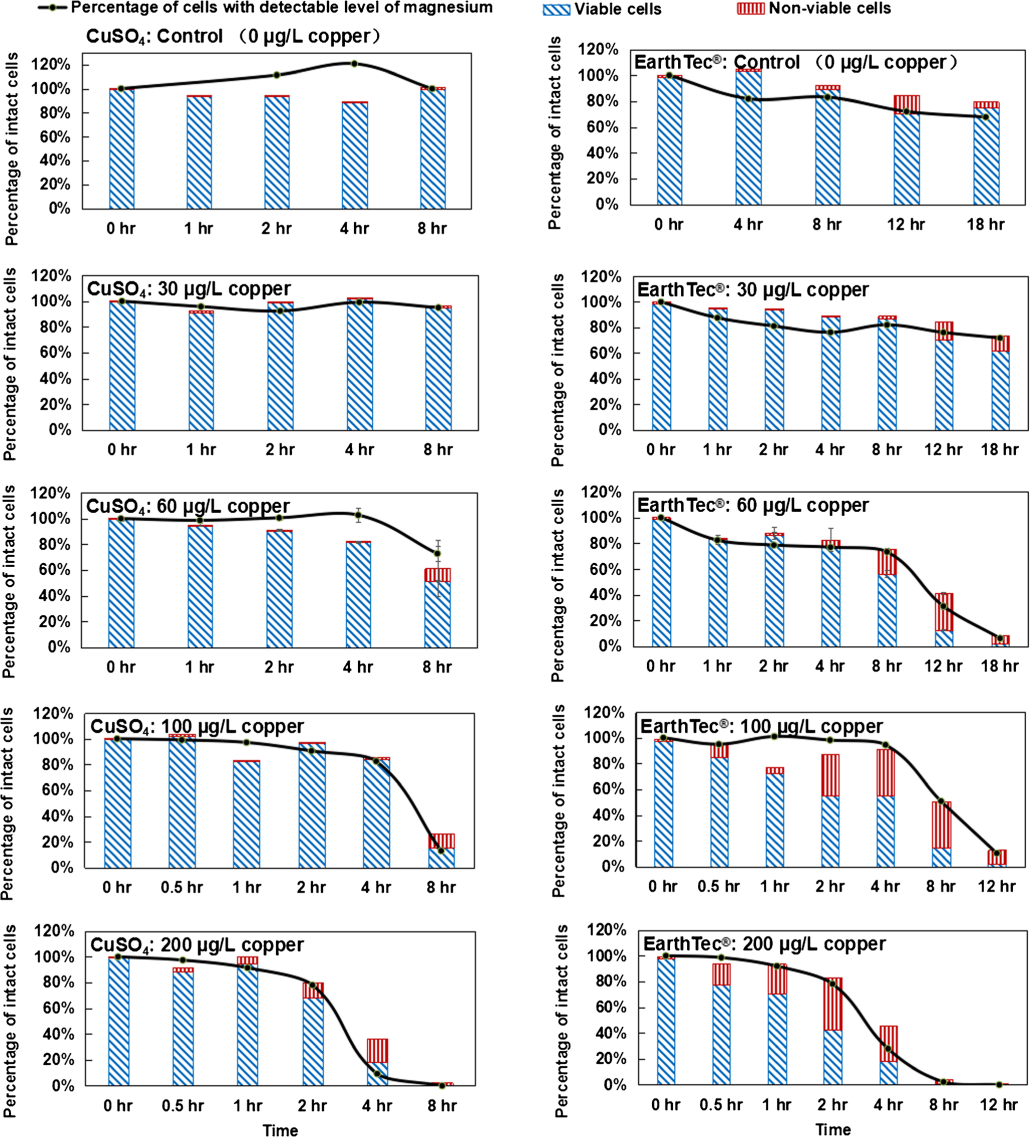
Cell viability and integrity following exposure to CuSO_4_ and EarthTec® over time using flow cytometry (bars) and SC-ICP-MS (lines), respectively

Flow cytometry is a technique capable of monitoring multiple characteristics of a single cell, and has the ability to distinguish cell debris and intact cells, as well as alive and dead cells [47]. In this study, flow cytometry was used to verify the copper-based algaecide effectiveness results obtained by the SC-ICP-MS method. First, the flow cytometer accuracy was determined and compared with that obtained from a hemocytometer using a fresh suspension of *M. aeruginosa* with a cell concentration of 1,000,000 cells/mL (all in triplicate). This comparison demonstrated that the flow cytometer arrived at a similar cellular concentration as the hemocytometer. Next, the accuracy of the SC-ICP-MS method to detect intact cells based on intrinsic magnesium was verified using flow cytometry which indicated cell integrity and viability for *M. aeruginosa* stained with nucleic acid dyes. An aliquot of cell sample was dyed with nucleic acid stains and then intact cell concentration and cell viability were detected by a flow cytometer when cell status was monitored by SC-ICP-MS. This comparison demonstrated that the SC-ICP-MS method accurately detected cell integrity following exposure to both cupric sulfate and EarthTec® (Fig. 3). Furthermore, this experiment revealed that the EarthTec® treatment resulted in both intact viable cells and intact nonviable cells whereas cupric sulfate treatment resulted primarily in intact viable cells. This difference implies that the treatment with EarthTec® resulted in slower cell lysis after killing the cells compared to cupric sulfate treatment.

### Determination of cellular copper uptake by SC-ICP-MS

First, the performance of the SC-ICP-MS was verified using the modified BG-11 medium. The matrix-matched calibration curve showed excellent linearity (*R*^2^ > 0.99) and water reference standard (CRM-TMDW-A) recoveries and algaecide spiked recoveries were in the range of 80% to 100%. Within the control groups for cupric sulfate and EarthTec® treatments, mean intracellular copper masses were 237 ± 15 ag/cell and 207 ± 14 ag/cell, respectively, representing 31% ± 3% and 49% ± 5% of *M. aeruginosa* cells with detectable levels of copper, respectively (ESM Fig. S3). This indicated that a low level of Cu was present in the *M. aeruginosa* before copper-based algaecide treatment. The difference between cupric sulfate and EarthTec® treatments may be attributed to the different batches of cells. It is known that copper is an essential element for organisms, and the original BG-11 medium contains 20 μg/L copper ions to support cell life activity, so only partial cells contained low and detectable levels of copper in the control groups, where cells were suspended in modified BG-11 medium without Cu. Though not all untreated cells contained copper, the cells took up much more copper after algaecide treatments; to highlight copper uptake in algaecide treatment groups, any measurements less than 237 ag/cell or 207 ag/cell for cupric sulfate and EarthTec® treatment groups, respectively, were excluded from mass distributions of algaecide treatment groups.

**Fig. 4:**
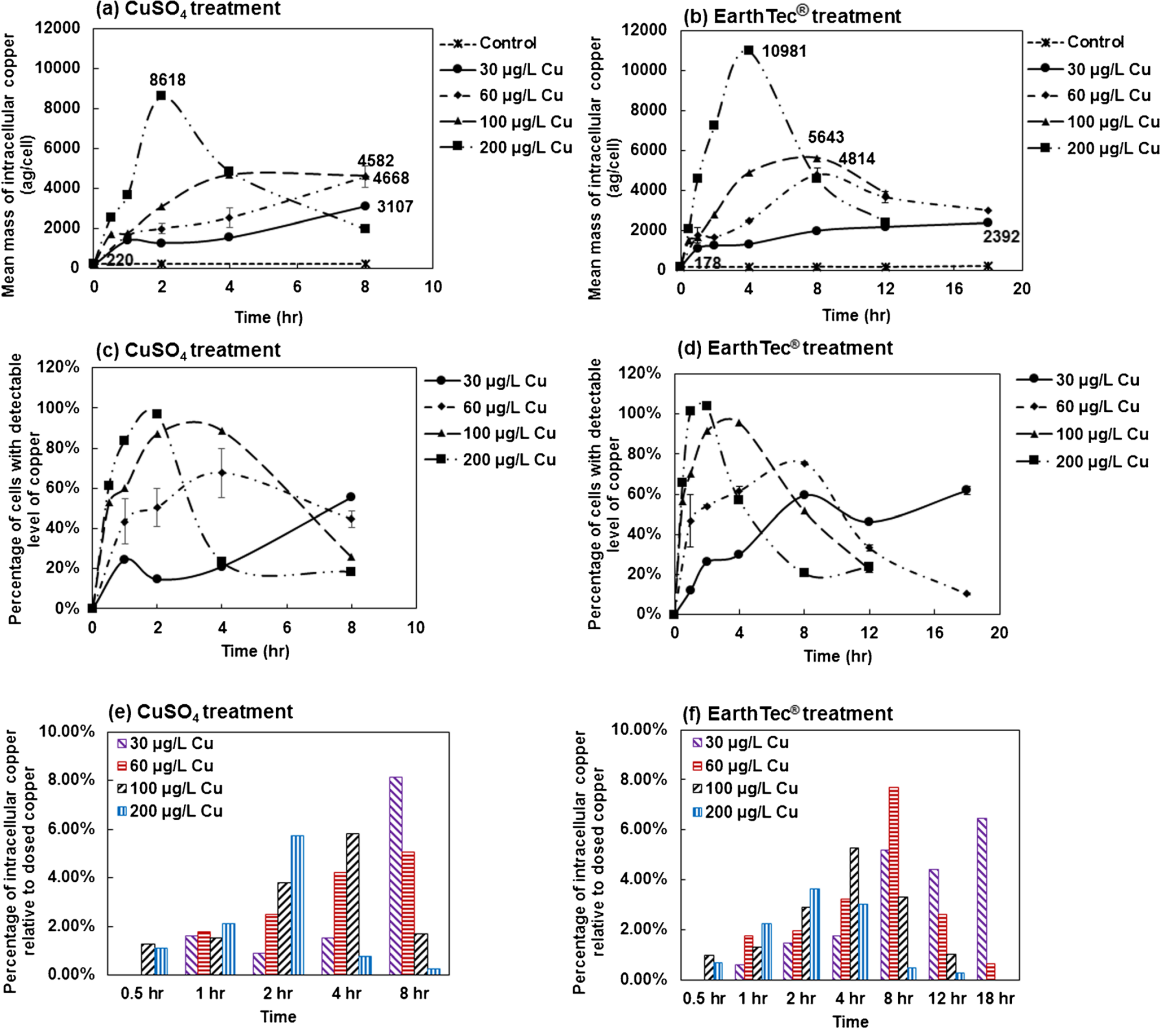
Intracellular copper levels following exposure to (**a)** CuSO_4_ and (**b)** EarthTec® over time, the percentage of cells with detectable levels of copper following exposure to ( **c)** CuSO_4_ and (**d)** EarthTec® over time, and the percentage of intracellular copper in *M. aeruginosa* relative to dosed copper following exposure to (**e)** CuSO_4_ and (**f)** EarthTec® over time

Our experiment suggested that *M. aeruginosa* lysed following hyperaccumulation of copper; *M. aeruginosa* cells uptake copper faster by cupric sulfate treatment than the EarthTec® treatment; *M. aeruginosa* cells uptake copper faster by higher algaecide concentration (Fig. 4). The mean intracellular copper mass increased slowly over 8 h in the 30, 60, and 100 μg/L copper doses in the cupric sulfate treatment groups. In contrast, the 200 μg/L dose quickly accumulated intracellular copper during the first 2 h, after which intracellular Cu decreased, suggesting that some of the cells were damaged and releasing Cu during lysis. (Fig. 4a). In the EarthTec® treatment groups, intracellular copper changed similarly with that of the cupric sulfate treatment groups over 8 h in the 30, 60, 100, and 200 μg/L copper treatments, followed by continual reductions in the higher-dosage groups (60, 100, and 200 μg/L copper), while continuously increasing in the 30 μg/L copper treatment group up to 18 h (Fig. 4b). Similarly, the number of cells with detectable amounts of copper also peaked and declined following increased exposure to Cu-EarthTec® (Fig. 4c, d), but at a slower speed and higher amount of Cu per cell before decreasing compared with the cupric sulfate treatment. Taken together, the exact intracellular copper content which is supposed to cause cell lysis and 100% of cells containing detectable copper were not displayed in all treatment groups. The results implied cell heterogeneity for *M. aeruginosa* during the copper treatment, i.e., cells that uptake copper fast would lyse once reaching a certain content of intracellular copper and cells that uptake copper slowly would stay alive and accumulate a higher amount of mass before lysing. The mean intracellular copper masses and the concentrations of cells containing detectable levels of copper were computed from those live cells.

**Fig. 5:**
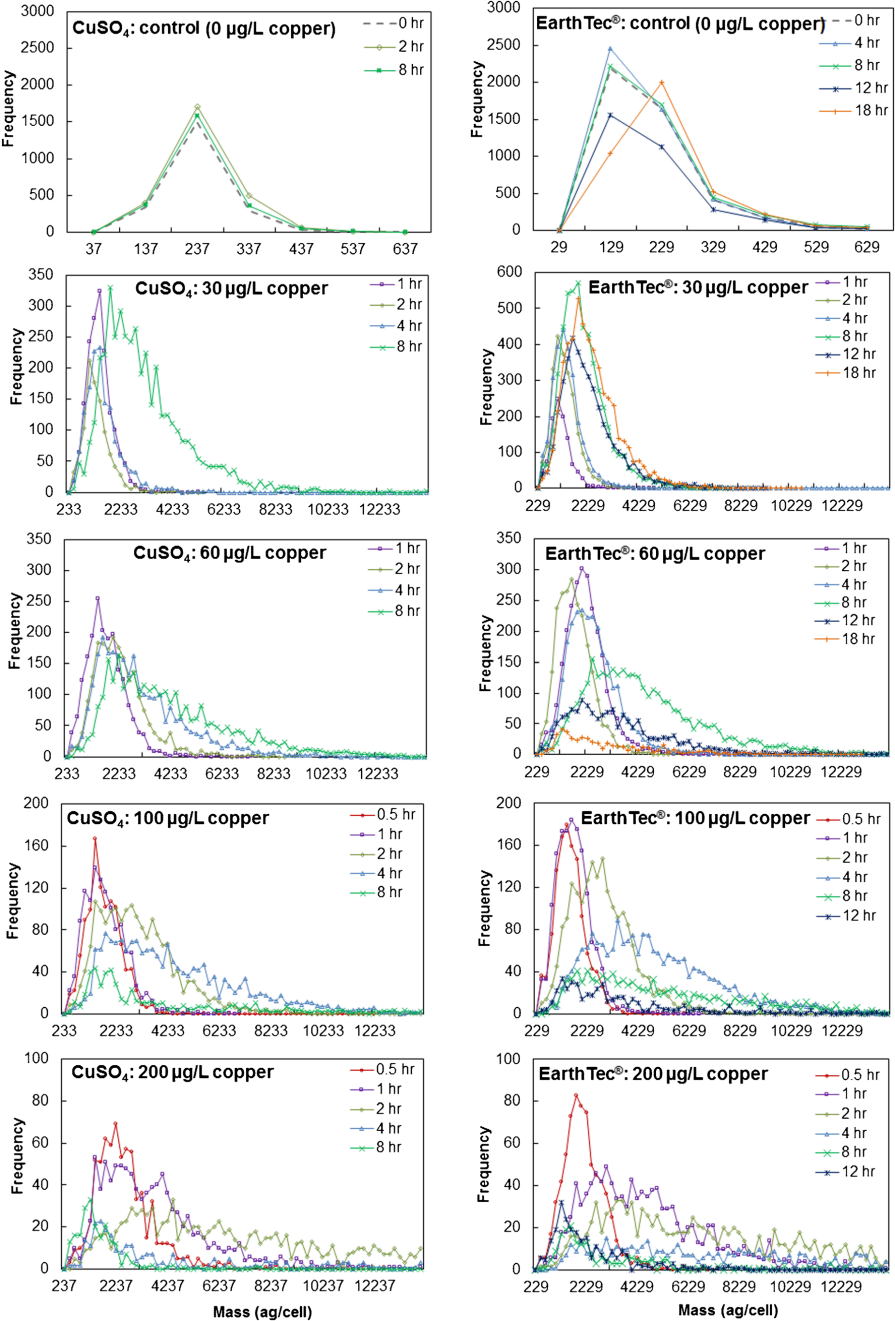
Copper mass distributions in cell populations treated with CuSO_4_ and EarthTec®

Treatment with cupric sulfate or EarthTec® both caused rapid accumulation of intracellular copper, as shown in Fig. 4. Higher doses of algaecide resulted in a more rapid cellular lysis, as previously discussed in regard to the magnesium release and flow cytometry measurements. Mass balance calculation showed that the highest copper uptake by cell populations during treatments, relative to the dosed concentrations, were 8.13%, 5.99%, 5.83%, and 5.72% copper ions in 30, 60, 100, and 200 μg/L copper of CuSO_4_ treatment groups, respectively, and 6.44%, 7.69%, 5.29%, and 3.64% copper ions in 30, 60, 100, and 200 μg/L copper of EarthTec® treatment groups, respectively (Fig. 4e, f), and these only represent a lesser percentage (within analytical deviation range) of dosed copper. Meanwhile, extracellular copper concentrations did not change significantly throughout the experiment, which was consistent with calculated copper uptake. The real-time signal also showed the extracellular copper concentration did not change significantly in treated groups after treatment for different times, such as 200 μg Cu/L of the EarthTec® treatment group (ESM Fig. S4), though the pulse signals that represent the copper taken by cells increased during the first 2 h and then decreased with the exposure time increasing, indicating the cells’ lysis. Therefore, our data suggests that excessive copper doses speed up copper uptake and cell lysis, rather than increases the amount of copper uptake. Notably, and based on the mass distribution observations (Fig. 5), *M. aeruginosa* cells exhibited marked heterogeneity in copper uptake from the surrounding matrix, corroborating the theory discussed above that there were differences among cells in copper uptake and cells lysed following hyperaccumulation of copper. The intracellular copper mass distributions were highly symmetrical prior to cell lysis; however, there were notable long tails in the mass distributions following more cells lysing (Fig. 5).

### Detection of MCs by UFLC-MS/MS

**Fig. 6:**
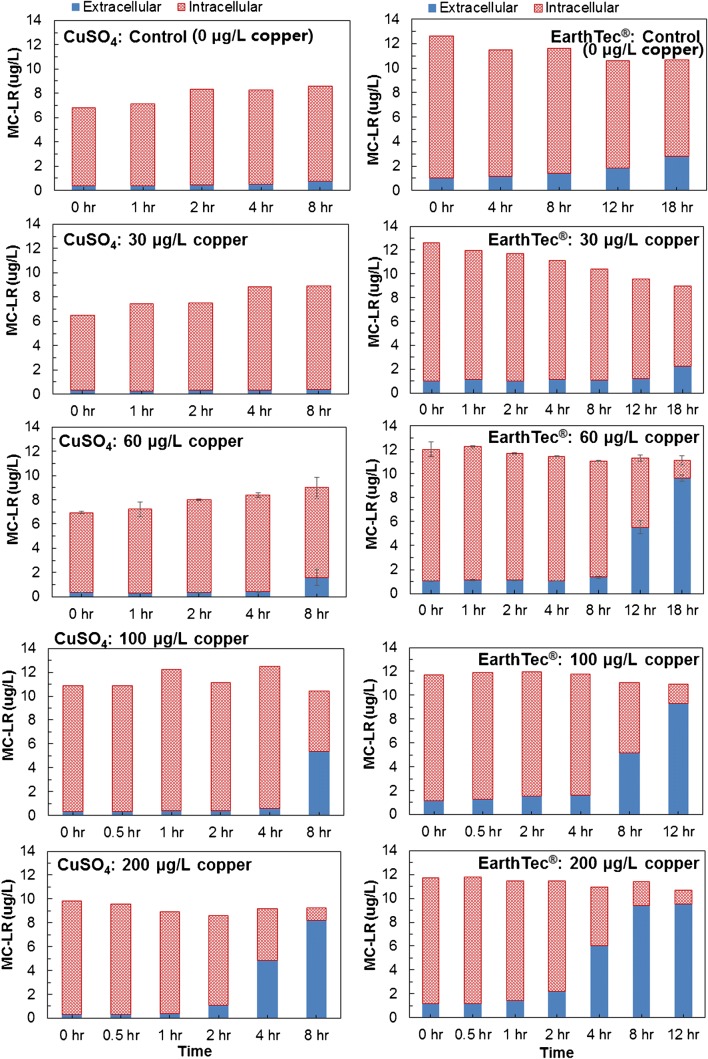
Intracellular and extracellular MC-LR concentrations following exposure to CuSO_4_ and EarthTec® over time

As another mode to assess the effectiveness of cupric sulfate and EarthTec® as preferred algaecides, the intracellular and extracellular levels of algal toxin MC-LR produced by *M. aeruginosa* were also monitored using the UFLC-MS/MS method [42]. These measurements were used to determine whether the algaecides would affect MC-LR and its release to the extracellular matrix. Consistent with our findings from the SC-ICP-MS and flow cytometry experiments, extracellular concentrations of MC-LR did increase following exposure to both algaecides. Specifically, extracellular MC-LR increased in a dose-dependent manner while total MC-LR concentrations remained unchanged (Fig. 6). In this way, treatment of *M. aeruginosa* with cupric sulfate and EarthTec® at sufficiently high doses invariably leads to cellular lysis. Therefore, such interventions are useful for controlling *M. aeruginosa* blooms, but fail to control the subsequent release of accumulated MCs into water. These results have implications in water treatment in which cellular lysis can inadvertently release MCs.

## Conclusions

In this study, a sensitive and rapid SC-ICP-MS method was developed to monitor cell status and quantify copper uptake by individual *M. aeruginosa* cells. The method was then applied to the study of two promising copper-based algaecides: cupric sulfate and a commercial copper-based product, EarthTec®. Our findings suggested that the SC-ICP-MS method was both sensitive and reproducible for the quantitation of copper in individual *M. aeruginosa* cells. Compared with other SC-ICP-MS methods, the developed method detected cell suspensions directly after suitable dilution, instead of repeat cell washings and re-suspensions, which avoided possible cell loss/damages and simplified sample preparation. The novel method for TE detection using a known concentration of fresh cell suspension as standard through detecting intrinsic magnesium of individual *M. aeruginosa* cells produces true TE. Treatments with 60, 100, and 200 μg/L copper as cupric sulfate and EarthTec® successfully diminished *M. aeruginosa* populations. The SC-ICP-MS method, alongside flow cytometry and UFLC-MS/MS measurements, suggested that these two algaecides led to copper hyperaccumulation followed by cellular lysis. Moreover, cell lysis or magnesium release in cupric sulfate treatment groups was faster than that in EarthTec® treatment groups. In both treatments, the higher the copper concentration the cells were treated with, the faster the copper uptake rate, and more cells lysed or magnesium released. However, further work will be needed to control the release of MCs into water since copper accumulation and subsequent lysis alone were not found to reduce MC-LR; thus, further treatment of this and other algal toxins possibly released together is needed.

## Electronic supplementary material

ESM 1 (PDF 382 kb)

## References

[1] Conley DJ, Paerl HW, Howarth RW, Boesch DF, Seitzinger SP, Havens KE (2009). Ecology. Controlling eutrophication: nitrogen and phosphorus. Science..

[2] O’neil J, Davis T, Burford M, Gobler C (2012). The rise of harmful cyanobacteria blooms: the potential roles of eutrophication and climate change. Harmful Algae.

[3] Harke MJ, Steffen MM, Gobler CJ, Otten TG, Wilhelm SW, Wood SA (2016). A review of the global ecology, genomics, and biogeography of the toxic cyanobacterium, *Microcystis* spp. Harmful Algae.

[4] Oberholster P, Botha A, Grobbelaar J. *Microcystis aeruginosa*: source of toxic microcystins in drinking water. Afr J Biotechnol. 2004;3(3).

[5] Zurawell RW, Chen H, Burke JM, Prepas EE (2005). Hepatotoxic cyanobacteria: a review of the biological importance of microcystins in freshwater environments. J Toxicol Environ Health B Crit Rev.

[6] Graham JL, Loftin KA, Meyer MT, Ziegler AC (2010). Cyanotoxin mixtures and taste-and-odor compounds in cyanobacterial blooms from the Midwestern United States. Environ Sci Technol.

[7] Takenaka S, Otsu R (2000). Effects of L-cysteine and reduced glutathione on the toxicities of microcystin LR: the effect for acute liver failure and inhibition of protein phosphatase 2A activity. Aquat Toxicol.

[8] Backer LC, Manassaram-Baptiste D, LePrell R, Bolton B (2015). Cyanobacteria and algae blooms: review of health and environmental data from the Harmful Algal Bloom-Related Illness Surveillance System (HABISS) 2007-2011. Toxins (Basel).

[9] Anderson DM (2009). Approaches to monitoring, control and management of harmful algal blooms (HABs). Ocean Coast Manag.

[10] Sengco MR, Anderson DM (2004). Controlling harmful algal blooms through clay flocculation 1. J Eukaryot Microbiol.

[11] Park CB, Baik S, Kim S, Choi JW, Lee SH, Kim Y (2017). The use of ultrasonic frequencies to control the bloom formation, regrowth, and eco-toxicity in *Microcystis aeruginosa*. Int J Environ Sci Technol.

[12] Visser PM, Ibelings BW, Bormans M, Huisman J (2016). Artificial mixing to control cyanobacterial blooms: a review. Aquat Ecol.

[13] Yu X, Cai G, Wang H, Hu Z, Zheng W, Lei X (2018). Fast-growing algicidal *Streptomyces* sp. U3 and its potential in harmful algal bloom controls. J Hazard Mater.

[14] Sun R, Sun P, Zhang J, Esquivel-Elizondo S, Wu Y (2018). Microorganisms-based methods for harmful algal blooms control: a review. Bioresour Technol.

[15] Waajen GW, Van Bruggen NC, Pires LMD, Lengkeek W, Lürling M (2016). Biomanipulation with quagga mussels (*Dreissena rostriformis bugensis*) to control harmful algal blooms in eutrophic urban ponds. Ecol Eng.

[16] Tsai KP (2015). Effects of two copper compounds on *Microcystis aeruginosa* cell density, membrane integrity, and microcystin release. Ecotoxicol Environ Saf.

[17] Tsai KP (2016). Management of target algae by using copper-based algaecides: effects of algal cell density and sensitivity to copper. Water Air Soil Pollut.

[18] Hadjoudja S, Vignoles C, Deluchat V, Lenain J-F, Le Jeune A-H, Baudu M (2009). Short term copper toxicity on *Microcystis aeruginosa* and *Chlorella vulgaris* using flow cytometry. Aquat Toxicol.

[19] Greenfield DI, Duquette A, Goodson A, Keppler CJ, Williams SH, Brock LM (2014). The effects of three chemical algaecides on cell numbers and toxin content of the cyanobacteria *Microcystis aeruginosa* and *Anabaenopsis* sp. Environ Manag.

[20] Bibak M, Hosseini SA (2013). Review ways to control harmful algal bloom (HAB). World J Fish Mar Sci.

[21] Deng C, Pan X, Wang S, Zhang D (2014). Cu^2+^ inhibits photosystem II activities but enhances photosystem I quantum yield of *Microcystis aeruginosa*. Biol Trace Elem Re.

[22] Bossuyt BT, Janssen CR (2004). Long-term acclimation of *Pseudokirchneriella subcapitata* (Korshikov) Hindak to different copper concentrations: changes in tolerance and physiology. Aquat Toxicol.

[23] Cerchiaro G, Manieri TM, Bertuchi FR (2013). Analytical methods for copper, zinc and iron quantification in mammalian cells. Metallomics..

[24] Merrifield RC, Stephan C, Lead JR (2018). Quantification of Au nanoparticle biouptake and distribution to freshwater algae using single cell - ICP-MS. Environ Sci Technol.

[25] Meyer S, López-Serrano A, Mitze H, Jakubowski N, Schwerdtle T (2018). Single-cell analysis by ICP-MS/MS as a fast tool for cellular uptake studies of arsenite. Metallomics..

[26] Ho KS, Chan WT (2010). Time-resolved ICP-MS measurement for single-cell analysis and on-line cytometry. J Anal Atom Spectrom.

[27] Zheng LN, Wang M, Wang B, Chen H-Q, Ouyang H, Zhao YL (2013). Determination of quantum dots in single cells by inductively coupled plasma mass spectrometry. Talanta..

[28] Li F, Armstrong DW, Houk RS (2005). Behavior of bacteria in the inductively coupled plasma: atomization and production of atomic ions for mass spectrometry. Anal Chem.

[29] Ikehata M, Woolcock J, Murphy ME, Merrifield R, Stephan C, Woodbridge O. Iron content measurement in individual bacterial cells using SC-ICP-MS. PerkinElmer, application note, 2018.

[30] Tsang CN, Ho KS, Sun H, Chan WT (2011). Tracking bismuth antiulcer drug uptake in single *Helicobacter pylori* cells. J Am Chem Soc.

[31] Mavrakis E, Sakellaraki E, Gaulier M, Riaudel A, Pergantis S, Lydakis Simantiris N. Arsenic accumulation in *Chlamydomonas reinhardtii* cells grown in As-contaminated media. 15th international conference on environmental science and technology. 2017.

[32] Groombridge AS, Miyashita S-i, Fujii S-i, Nagasawa K, Okahashi T, Ohata M (2013). High sensitive elemental analysis of single yeast cells (*Saccharomyces cerevisiae*) by time-resolved inductively-coupled plasma mass spectrometry using a high efficiency cell introduction system. Anal Sci.

[33] Wang H, Wang B, Wang M, Zheng L, Chen H, Chai Z (2015). Time-resolved ICP-MS analysis of mineral element contents and distribution patterns in single cells. Analyst..

[34] Wang H, Wang M, Wang B, Zheng L, Chen H, Chai Z (2017). Interrogating the variation of element masses and distribution patterns in single cells using ICP-MS with a high efficiency cell introduction system. Anal Bioanal Chem.

[35] Wang H, Chen B, He M, Hu B (2017). A facile droplet-chip-time-resolved inductively coupled plasma mass spectrometry online system for determination of zinc in singlecCell. Anal Chem.

[36] Zheng LN, Wang M, Zhao LC, Sun BY, Wang B, Chen HQ (2015). Quantitative analysis of Gd@C82(OH)22 and cisplatin uptake in single cells by inductively coupled plasma mass spectrometry. Anal Bioanal Chem.

[37] Yu X, Chen B, He M, Wang H, Hu B (2018). Chip-based magnetic solid phase microextraction coupled with ICP-MS for the determination of Cd and Se in HepG2 cells incubated with CdSe quantum dots. Talanta..

[38] Corte Rodriguez M, Alvarez-Fernandez Garcia R, Blanco E, Bettmer J, Montes-Bayon M (2017). Quantitative evaluation of cisplatin uptake in sensitive and resistant individual cells by single-cell ICP-MS (SC-ICP-MS). Anal Chem.

[39] Oliver AL-S, Baumgart S, Bremser W, Flemig S, Wittke D, Grützkau A (2018). Quantification of silver nanoparticles taken up by single cells using inductively coupled plasma mass spectrometry in the single cell measurement mode. J Anal Atom Spectrom.

[40] Zeng J, Yang L, Wang WX (2009). Acclimation to and recovery from cadmium and zinc exposure by a freshwater cyanobacterium, *Microcystis aeruginosa*. Aquat Toxicol.

[41] Zeng J, Zhao D, Ji Y, Wu Q (2012). Comparison of heavy metal accumulation by a bloom-forming cyanobacterium, *Microcystis aeruginosa*. Chin Sci Bull.

[42] Zhang H, Dan Y, Adams CD, Shi H, Ma Y, Eichholz T (2017). Effect of oxidant demand on the release and degradation of microcystin-LR from *Microcystis aeruginosa* during oxidation. Chemosphere..

[43] Ding J, Shi H, Timmons T, Adams C (2009). Release and removal of microcystins from microcystis during oxidative-, physical-, and UV-based disinfection. J Environ Eng.

[44] Dan Y, Zhang W, Xue R, Ma X, Stephan C, Shi H (2015). Characterization of gold nanoparticle uptake by tomato plants using enzymatic extraction followed by single-particle inductively coupled plasma–mass spectrometry analysis. Environ Sci Technol.

[45] Montaser A (1998). Inductively coupled plasma mass spectrometry.

[46] Pace HE, Rogers NJ, Jarolimek C, Coleman VA, Higgins CP, Ranville JF (2011). Determining transport efficiency for the purpose of counting and sizing nanoparticles via single particle inductively coupled plasma mass spectrometry. Anal Chem.

[47] Adan A, Alizada G, Kiraz Y, Baran Y, Nalbant A (2017). Flow cytometry: basic principles and applications. Crit Rev Biotechnol.

