# Supplementary material for: Evaluating the treatment effectiveness of copper-based algaecides on toxic algae Microcystis aeruginosa using single cell-inductively coupled plasma-mass spectrometry
Source: Anal Bioanal Chem. 2019 Jun 14;411(21):5531–43. doi: 10.1007/s00216-019-01933-9 (PMC6684568; doi:10.1007/s00216-019-01933-9)
Supplement: Supplementary file 1 — (PDF 382 kb) [file 216_2019_1933_MOESM1_ESM.pdf]

Analytical and Bioanalytical Chemistry

Electronic Supplementary Material

Evaluating the treatment effectiveness of copper-based algaecides on toxic algae *Microcystis aeruginosa* using single cell – inductively coupled plasma – mass spectrometry

Xing Shen, Haiting Zhang, Xiaolong He, Honglan Shi, Chady Stephan, Hua Jiang, Cuihong Wan, Todd Eichholz

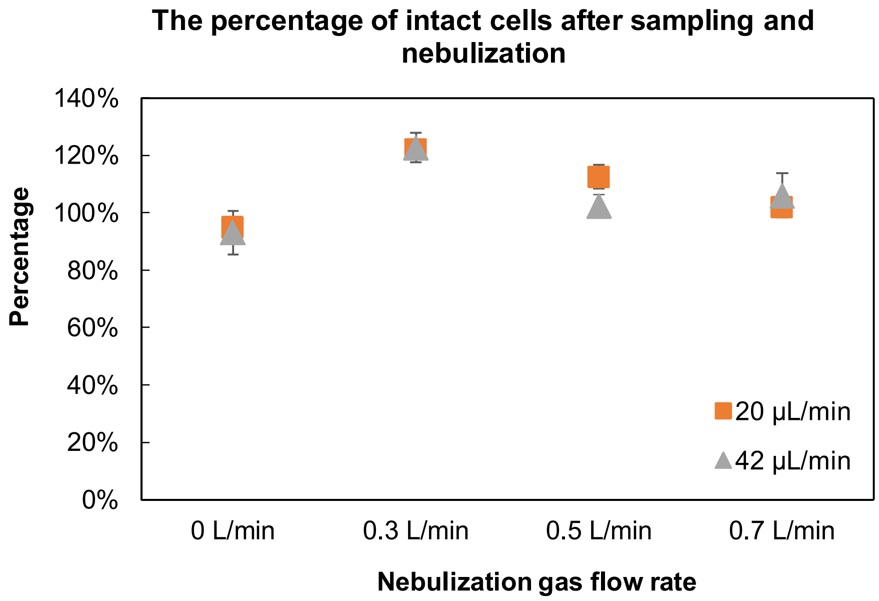

Fig. S1 The concentrations of intact cells following nebulization with variable nebulization gas flow rates (0, 0.3, 0.5, and 0.7 L/min) and sample flow rate (20 μL/min and 42 μL/min)

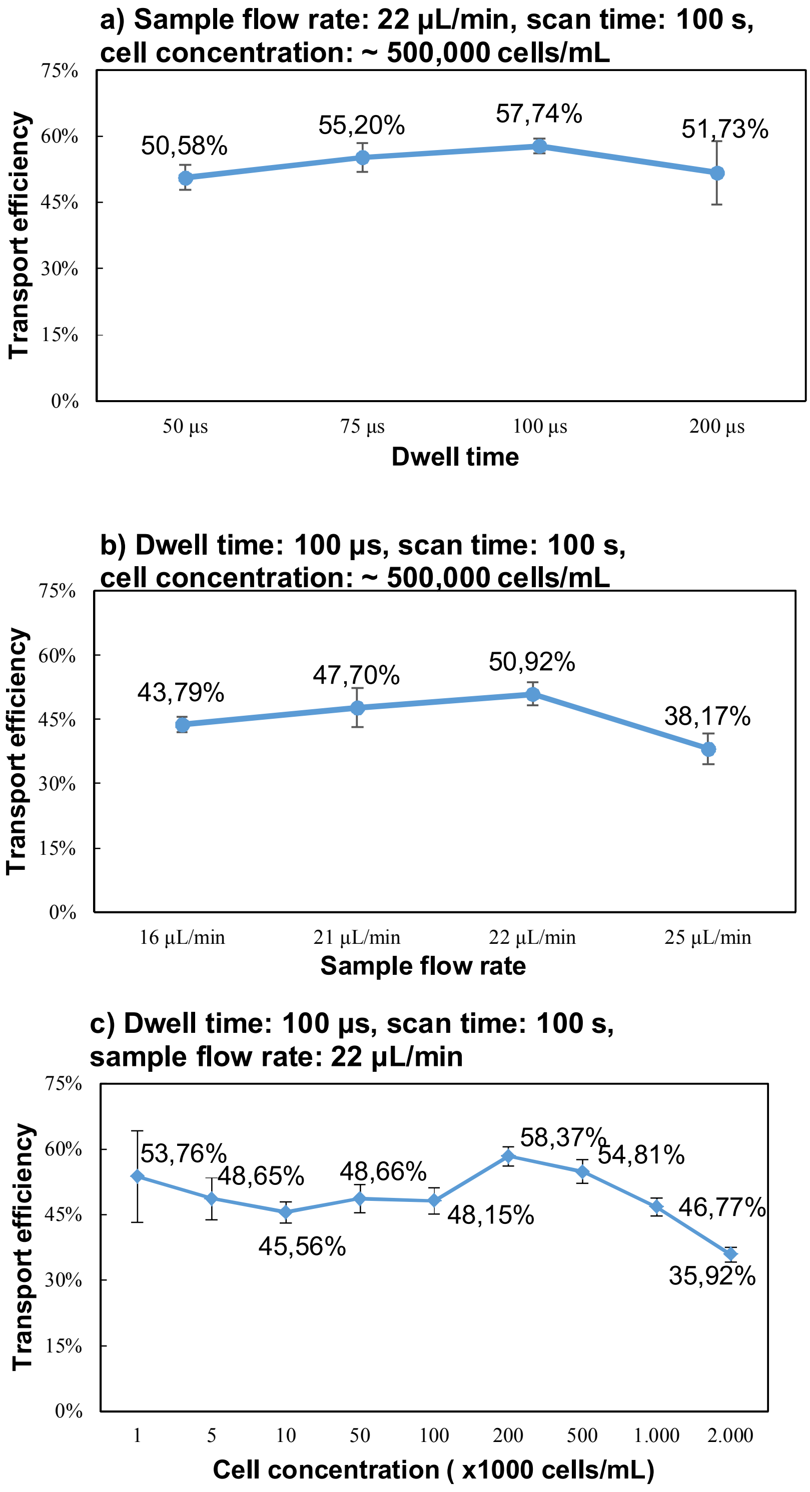

Fig. S2 Transport efficiencies for *M. aeruginosa* cells with variable (a) dwell times, (b) sample flow rates, and (c) cell concentrations

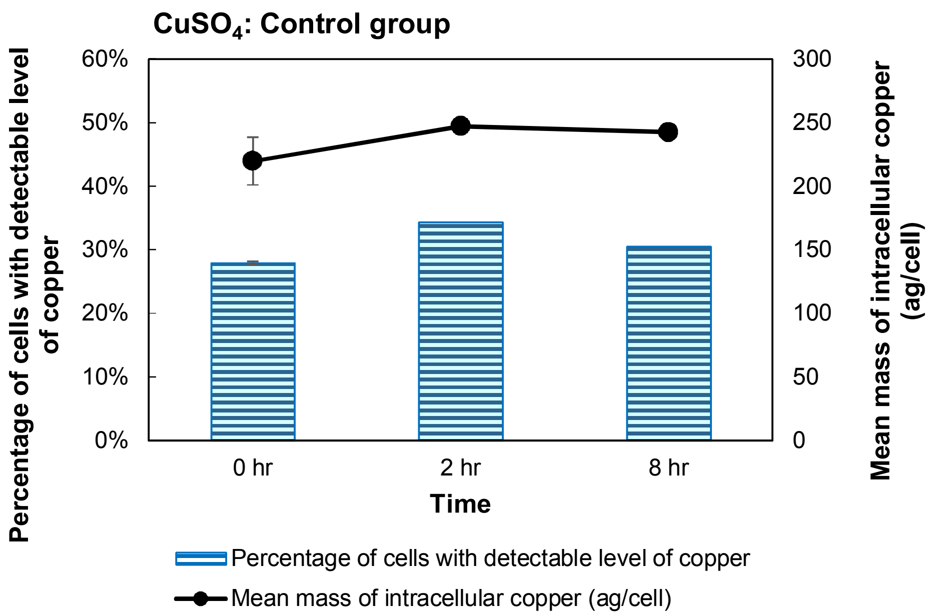

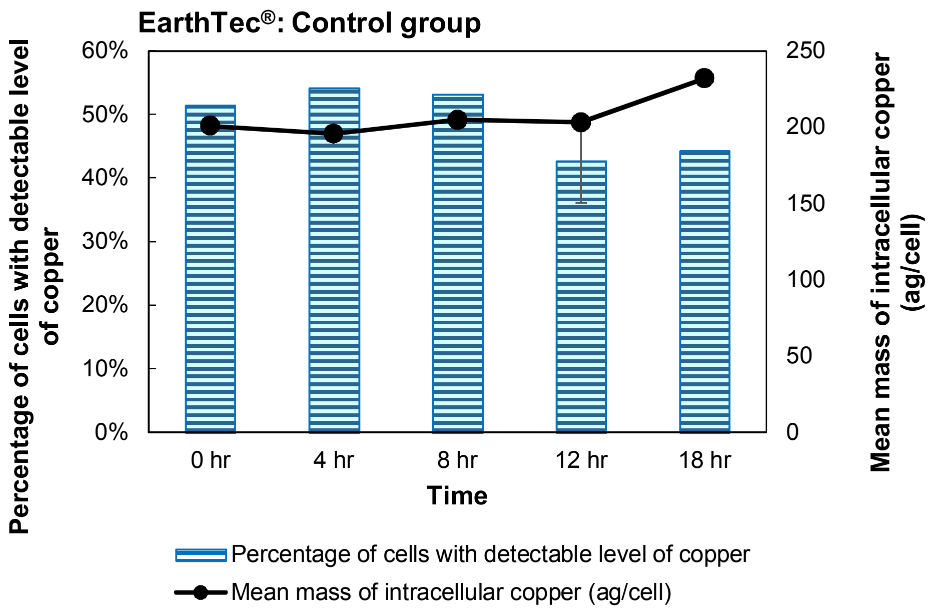

Fig. S3 The percentage of cells with detectable level of copper and mean copper mass per cell in control groups (without algaecide addition)

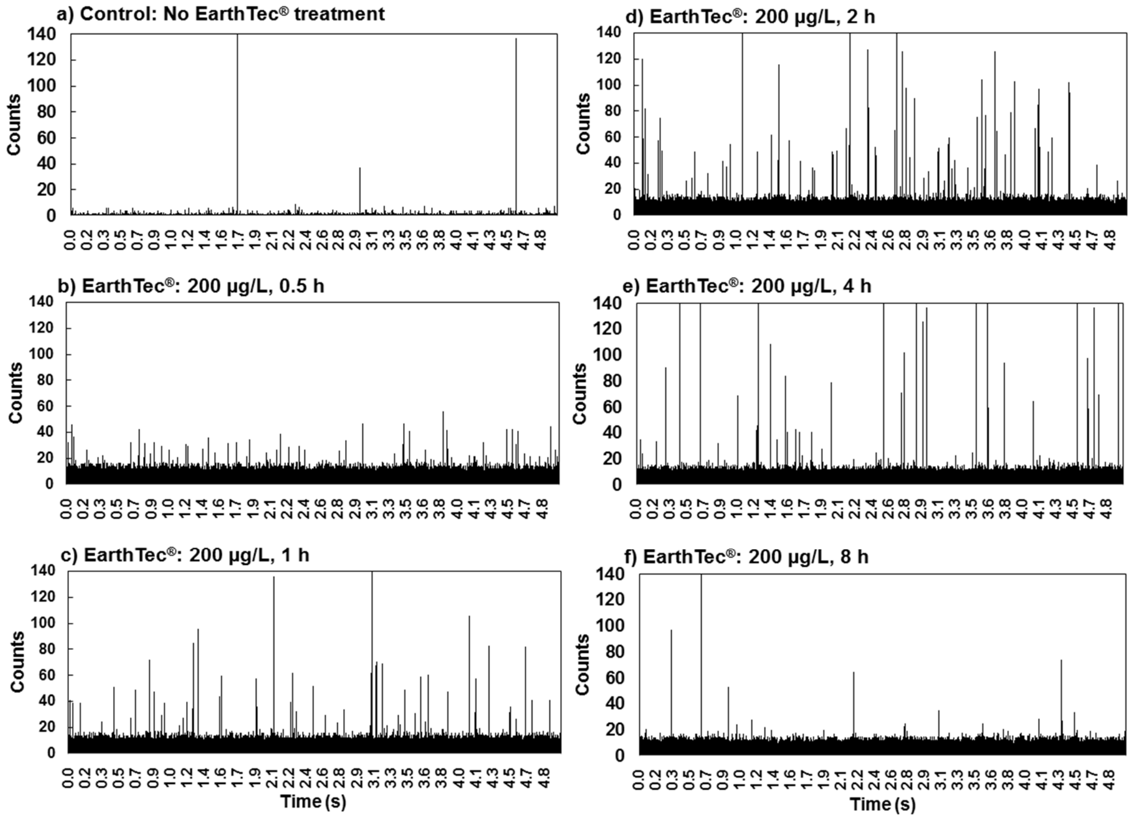

Fig. S4 Raw data for the cells without exposure to EarthTec® (a) during the first 5 s of scanning; and raw data for the cells exposure to 200 µg Cu/L of EarthTec® for 0.5 h (b) , 1 h (c), 2 h (d), 4 h (e) and 8 h (f) during the first 5 s of scanning
